# Expression of EhRAD54, EhRAD51, and EhBLM proteins during DNA repair by homologous recombination in *Entamoeba histolytica*

**DOI:** 10.1051/parasite/2014006

**Published:** 2014-02-19

**Authors:** Ma. del Socorro Charcas-Lopez, Lorena Garcia-Morales, Marisol Pezet-Valdez, Cesar Lopez-Camarillo, Absalom Zamorano-Carrillo, Laurence A. Marchat

**Affiliations:** 1 Programa Institucional de Biomedicina Molecular, Escuela Nacional de Medicina y Homeopatía del IPN, Guillermo Massieu Helguera No. 239, Fracc. La Escalera, Ticoman México D.F. C.P. 07320 Mexico; 2 Posgrado en Ciencias Genómicas, Universidad Autónoma de la Ciudad de México, San Lorenzo No. 290, Col. del Valle México D.F. C.P. 03110 Mexico; 3 Programa de Doctorado en Biotecnología, Escuela Nacional de Medicina y Homeopatía del IPN, Guillermo Massieu Helguera No. 239, Fracc. La Escalera, Ticoman México D.F. C.P. 07320 Mexico

**Keywords:** DNA double-strand break repair, Homologous recombination, Amoebiasis

## Abstract

*Entamoeba histolytica*, the protozoan responsible for human amoebiasis, exhibits a great genome plasticity that is probably related to homologous recombination events. It contains the RAD52 epistasis group genes, including *Ehrad51* and *Ehrad54*, and the *Ehblm* gene, which are key homologous recombination factors in other organisms. *Ehrad51* and *Ehrad54* genes are differentially transcribed in trophozoites when DNA double-strand breaks are induced by ultraviolet-C irradiation. Moreover, the EhRAD51 recombinase is overexpressed at 30 min in the nucleus. Here, we extend our analysis of the homologous recombination mechanism in *E. histolytica* by studying EhRAD51, EhRAD54, and EhBLM expression in response to DNA damage. Bioinformatic analyses show that EhRAD54 has the molecular features of homologous proteins, indicating that it may have similar functions. Western blot assays evidence the differential expression of EhRAD51, EhRAD54, and EhBLM at different times after DNA damage, suggesting their potential roles in the different steps of homologous recombination in this protozoan.

## Abstract (fr)

Le génome d’*Entamoeba histolytica,* le protozoaire parasite responsable de l’amibiase humaine, présente une grande plasticité qui est probablement associée à l’existence d’évènements de recombinaison homologue. Il possède les gènes du groupe d’épistasie RAD52, y compris les gènes *Ehrad51* et *Ehrad54*, ainsi que le gène *Ehblm*, qui sont les principaux facteurs de recombinaison homologue chez d’autres organismes. Les gènes *Ehrad51* et *Ehrad54* sont transcrits différemment chez les trophozoïtes lorsque des cassures double-brin de l’ADN sont induites par le rayonnement ultraviolet C. En outre, il y a une surexpression de la recombinase EhRAD51 dans le noyau après 30 minutes. Dans cet article, nous continuons notre analyse du mécanisme de recombinaison homologue chez *E. histolytica* en étudiant l’expression des protéines EhRAD51, EhRAD54 et EhBLM en réponse au dommage de l’ADN. Les analyses bioinformatiques montrent que EhRAD54 possède les caractéristiques moléculaires des protéines homologues, ce qui indique qu’elle pourrait avoir les mêmes fonctions. Les essais de Western blot montrent que les protéines EhRAD54, EhRAD51 et EhBLM s’expriment de façon différentielle à différents moments après le dommage de l’ADN, ce qui suggère leur rôle potentiel dans les différentes étapes de la recombinaison homologue chez ce protozoaire.

## Introduction

*Entamoeba histolytica* is the protozoan causative of human amoebiasis, a neglected parasitic disease that affects about 50 million people worldwide [29]. *E. histolytica* trophozoites show a dramatic virulence variability that has been related to a great genome plasticity, with ploidy changes, unscheduled gene amplification, and duplication events, which might be associated with genetic rearrangements mediated by homologous recombination (HR) events [1, 17, 32]. HR is the evolutionarily conserved pathway that promotes the restart of collapsed replication forks, faithful chromosome segregation, telomere maintenance, and repair of DNA double-strand breaks (DSBs) [13, 16, 31]. In eukaryotes, HR is carried out by members of the RAD52 epistasis group and additional proteins. Among them, RAD51, RAD54, and BLM proteins are key factors for the realization of the different steps of the HR process. RAD51 is the central recombinase, which catalyzes strand transfer between a broken DNA and its undamaged homologous strand, allowing the damaged region to be repaired [2, 22, 25]. The RAD54 translocase is a dsDNA-dependent ATPase of the Snf2/Swi2 family of SF2 helicases, although it lacks classical helicase activity [9, 10]. RAD54 interacts with RAD51 and it has been implicated in nearly all mechanistic stages of HR, including chromatin remodeling, RAD51-ssDNA filament stability, homology search and DNA strand invasion, D-loop dissolution and branch migration, dissociation of RAD51 from heteroduplex DNA to allow extension of the invading 3′-OH end by DNA polymerase, and turnover of RAD51-dsDNA dead-end complexes [4, 6, 24]. BLM is a multifunctional RecQ DNA helicase of the SF2 family that has both DNA-stimulated ATPase and ATP-dependent DNA helicase activities with a 3′–5′ polarity [11]. Interacting with RAD51 and RAD54 proteins, BLM can accurately control chromatin remodeling and RAD51 nucleofilament disruption in the synaptic phase [12, 21, 30]. It also stimulates strand exchange carried out by RAD51 [3, 23]. In addition, BLM can catalyze branch migration of Holliday junctions, unwind D-loops, and promote regression of model replication forks [18, 20].

We previously reported that *E. histolytica* has genes encoding putative EhRAD52 epistasis group members, which participate in recombinational DNA repair in other organisms, including the EhRAD51 recombinase and the EhRAD54 translocase [14], as well as a putative EhBLM protein [5]. The transcriptional profile of the RAD52 epistasis group-related genes evidenced the absence of a coordinated transcriptional activation in response to DNA damage induced by UV-C irradiation, suggesting that trophozoites have enough stationary levels of enzymes to perform the HR process that is essential for genome maintenance and survival. Interestingly, the amount of *Ehrad51* mRNA was about 15-fold higher at 30 min post-UV-C treatment and decreased 3 and 12 h later. Congruently, Western blot assays showed a dramatic increase in EhRAD51 protein in the nucleus at 30 min after DNA damage, which supports the relevance of the recombinase EhRAD51 in DNA repair by HR [14]. On the other hand, cDNA microarray experiments revealed that the *Ehrad54* mRNA level was increased at 5 min [28], which indicates that EhRAD54 may be involved early in HR. In order to contribute to the knowledge of the molecular events underlying DNA repair by HR in *E. histolytica*, here we evaluated the expression of EhRAD51, EhRAD54, and EhBLM proteins in the early response to DNA damage in this deep-branching eukaryotic parasite.

## Materials and methods

### *In silico* analysis

The predicted amino acid sequence corresponding to the *Ehrad54* gene was compared with homologous proteins from other organisms by BLAST and aligned with human RAD54 by ClustalW (http://www.ch.embnet.org/software/ClustalW.html) allowing gap penalties of 10 to maximize protein homology. Conserved domains were identified by the ScanProsite (http://us.expasy.org/tools/scanprosite/), MotifScan (http://myhits.isb-sib.ch/cgi-bin/motif_scan), and Pfam (http://www.sanger.ac.uk/Software/Pfam/search.shtml) programs. The 3D structure of EhRAD54 was predicted using crystallographic data of *Solfolobus solfataricus* ATPase SNF2 (PDB 1z6A) with the Swiss-Model software (http://www.expasy.ch/swissmod/), and visualized through the PyMol (http://pymol.sourceforge.net/) program.

### Peptide design and generation of specific antibodies

The predicted amino acid sequences of EhRAD54 and EhBLM proteins were analyzed by bioinformatic tools from Harvard University (http://mif.dfci.harvard.edu/tools/antigenic.pl) to identify antigenic determinants that were then submitted to BLAST to select peptides that only match with the target proteins. Their localization in the 3D model of each protein was determined by the LOOPP (http://cbsuapps.tc.cornell.edu/loopp.aspx) and PyMol programs.

The selected EhRAD54-pepA coupled to a system of multiple antigenicity (MAP) of eight asymmetric branches was synthesized by Genemed Synthesis Inc. (www.genemedsyn.com) and used as an antigen to generate the EhRAD54 antibody in mice. Briefly, EhRAD54-pepA (100 μg) mixed with TiterMax Gold adjuvant (50 μL) was intramuscularly inoculated (three times at 10-day intervals) into five pathogen-free BALB/c mice. At day 30, the animals were bled and serum was collected. The selected EhBLM-pepB peptide and rabbit antibody against EhBLM-pepB were purchased from GL Biochem (Shanghai) Ltd. Both immune sera were aliquoted and stored at −20 °C until use.

### Induction of DNA damage in *E. histolytica* trophozoites

*E. histolytica* HM-1:IMSS strain trophozoites axenically cultured in TYI-S-33 medium [7] at 37 °C were irradiated with 254 nm UV-C light (150 J/m^2^) for 8 s using a UV Stratalinker 1800 device (Stratagene), and incubated in fresh TYI-S-33 culture medium at 37 °C for 5 and 30 min after genotoxic treatment before being harvested. Induction of DSBs in this DNA damage model has been previously corroborated by evaluation of the EhH2AX histone phosphorylation status, and TUNEL and Comet assays [14].

### Preparation of cytoplasmic and nuclear protein extracts

Cytoplasmic (CE) and nuclear (NE) extracts from trophozoites were prepared following the protocol of Schreiber et al. [19] with some modifications [15]. Briefly, trophozoites (10^7^) were harvested and washed with cold PBS pH 6.8. Cell lysis was induced by incubation in four volumes of Buffer A (10 mM HEPES, pH 7.9, 1.5 mM MgCl_2_, 10 mM KCl, 0.5 mM DTT, 0.5 mM PMSF) containing protease inhibitors (0.5 mM PMSF; 2 mM benzamidine; 5 μg/mL of each aprotinin, pepstatin A, leupeptin, and E-64) at 4 °C for 20 min, monitoring nucleus integrity by phase-contrast microscopy. Then, samples were centrifuged at 14,000 rpm for 1 min to collect the supernatant corresponding to CE. The pellet containing nuclei was incubated for 40 min at 4 °C in 50 μL Buffer C (20 mM HEPES, pH 7.9, 0.42 mM NaCl, 1 mM EDTA, 1 mM PMSF, 1 mM EGTA, 0.5 mM DTT) in the presence of protease inhibitors, and centrifuged at 14,000 rpm for 5 min at 4 °C, to obtain the supernatant corresponding to NE. Both CE and NE were stored at −70 °C until use.

### Western blot assay

Cytoplasmic (CE) and nuclear extracts (NE) from irradiated and non-irradiated trophozoites were submitted to 10% SDS-PAGE and Coomassie blue staining to confirm their integrity. Then, proteins (25 μg/lane) were electrotransferred to a nitrocellulose membrane that was incubated with anti-EhRAD51 [14], anti-EhRAD45-pepA (1:500) or anti-EhBLM-pepB (1/1000) in 5% non-fat dry milk, and 0.05% Tween-20 in PBS pH 7.4 overnight at 4 °C. Anti-actin antibody (1:1000) was used as a control. Pre-immune sera were also tested as a control. Proteins were detected by peroxydase conjugated secondary goat antibodies (Zymed) and revealed by the ECL-Plus system (Amersham). Developed films were scanned and images were acquired by a gel documentation system (DNR Bio Imaging Systems Ltd.). Bands were submitted to densitometry analysis using the Gel Quant Express software. The background signal corresponding to a clear area in the same image was removed from the data of each protein. Pixels corresponding to cytoplasmic actin were taken as 100% at each time and used to normalize data. Finally, the relative expression of each protein was expressed using the amount of protein before UV-C exposure as a reference.

## Results and discussion

**Figure 1.**
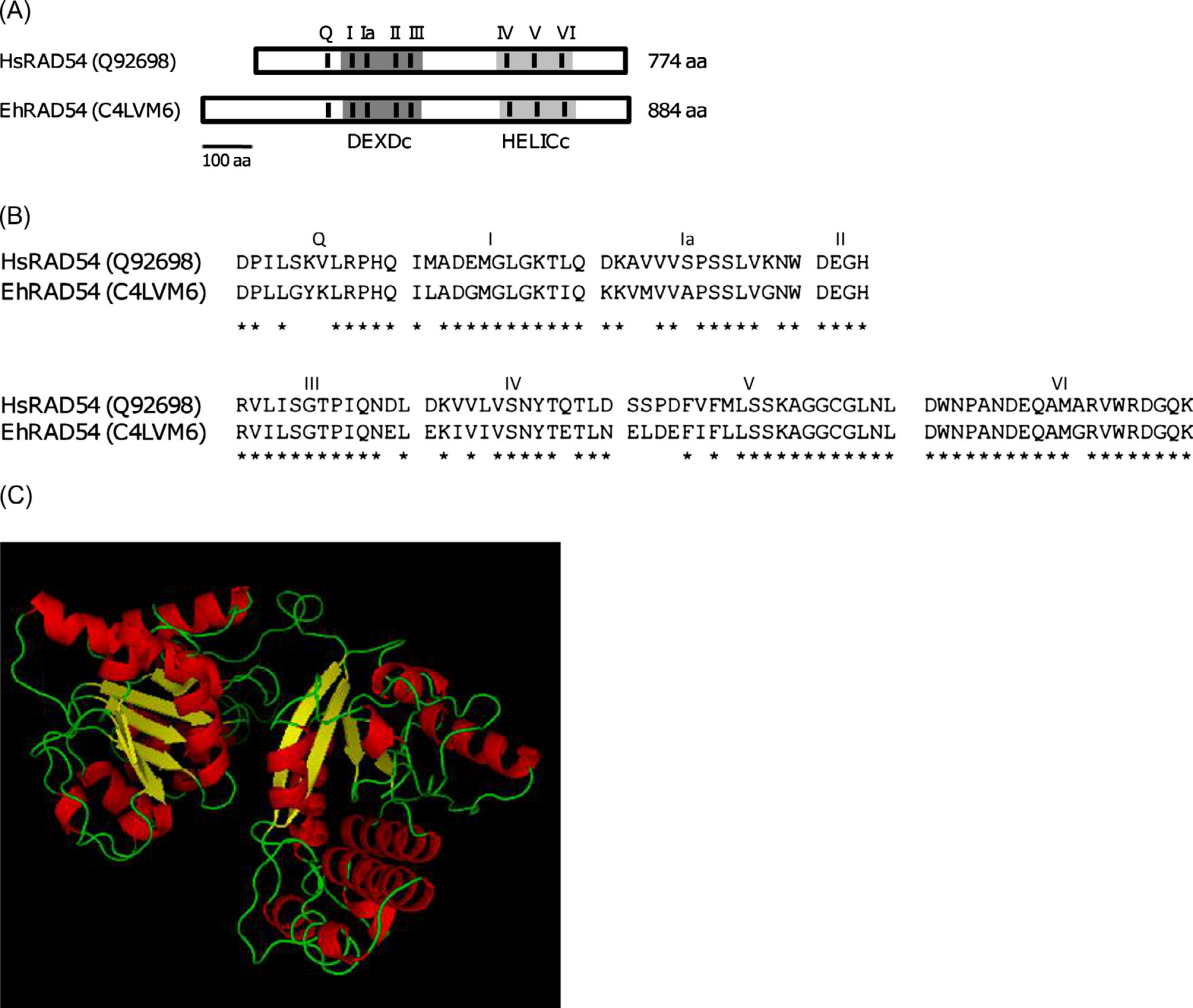
Molecular characteristics of EhRAD54 in *E. histolytica* (Eh) and *Homo sapiens* (Hs). (A) Schematic representation. Numbers at the right indicate the size in amino acids (aa) for each protein. The scale is at the bottom. (B) Alignment of conserved motifs. Asterisks indicate identical aa. (C) Predicted 3D model for EhRAD54. Accession number in UniProt Knowledgebase (UniProtKB) is indicated for each protein.

The recombinase RAD51, the translocase RAD54, and the helicase BLM are conserved proteins that act in a coordinated way in the different steps of the homologous recombination (HR) pathway to maintain genomic stability of eukaryotic cells [4]. We previously reported that predicted amino acid sequences of *E. histolytica* EhRAD51 and EhBLM proteins have the molecular characteristics of homologous proteins described in other organisms [5, 14]. Here, we show that the intronless *Ehrad54* gene (2655 bp) corresponds to an 884 aa (100 kDa) polypeptide, which shares 54–61% similarity and 37–43% identity (e-values from e-176 to e-123) with homologous proteins in various eukaryotic organisms, from plant (*Arabidopsis thaliana*) to human, including other protozoan parasites, such as *Trypanosoma brucei* (Table 1). Analysis of the predicted EhRAD54 protein evidenced the presence of conserved motor domains known as DEXDc (291-462 aa) and HELICc (613-772 aa). These domains contain motifs I, Ia, II, and III, and motifs IV, V, and VI, that are necessary for protein activity in humans [9, 10]. At the amino terminus, EhRAD54 also has the Q motif that is important for stimulation of chromatin remodeling by RAD51 (Figure 1A and B) [27]. The 3D model of the EhRAD54 protein shows that the EhRAD54 core consists of two *α*/*β*-lobes with specific motifs that form a helical domain within each lobe (Figure 1C), as described for the crystallographic structures of the *S. solfataricus* [8] and zebra fish Rad54 [26] proteins. Notably, the broad cleft between the two lobes could be involved in DNA binding by SF2 helicases. The stability of the predicted 3D structure was validated by a Ramachandran plot and its similarity with the crystallographic structure used for prediction (PDB 1z6A) was confirmed by the RMSD value (1.903 Å) obtained from overlapping both structures (data not shown). Taken altogether, these data predicted that EhRAD54 conserves the molecular characteristics of its human homologue, which suggests that it could be participating in HR in *E. histolytica*, probably in coordination with other HR factors, such as EhRAD51 and EhBLM proteins.

**Table 1.** Comparison of predicted EhRAD54 with homologous proteins in other organisms.

Organism	Accession number*	E-value	Similarity (%)	Identity (%)
*Dictyostelium discoideum*	Q54RP8	e-176	59	42
*Gallus gallus*	Q9DG67	e-134	54	37
*Trypanosoma brucei*	Q385M5	e-132	59	43
*Danio rerio*	Q7ZV09	e-131	60	43
*Homo sapiens*	Q92698	e-127	59	42
*Xenopus laevis*	Q6INQ9	e-128	54	37
*Drosophila melanogaster*	O76460	e-125	61	41
*Caenorhabditis elegans*	G5EEN6	e-125	59	39
*Arabidopsis thaliana*	Q0PCS3	e-123	60	43

* UniProt Knowledgebase (UniProtKB).

**Figure 2.**
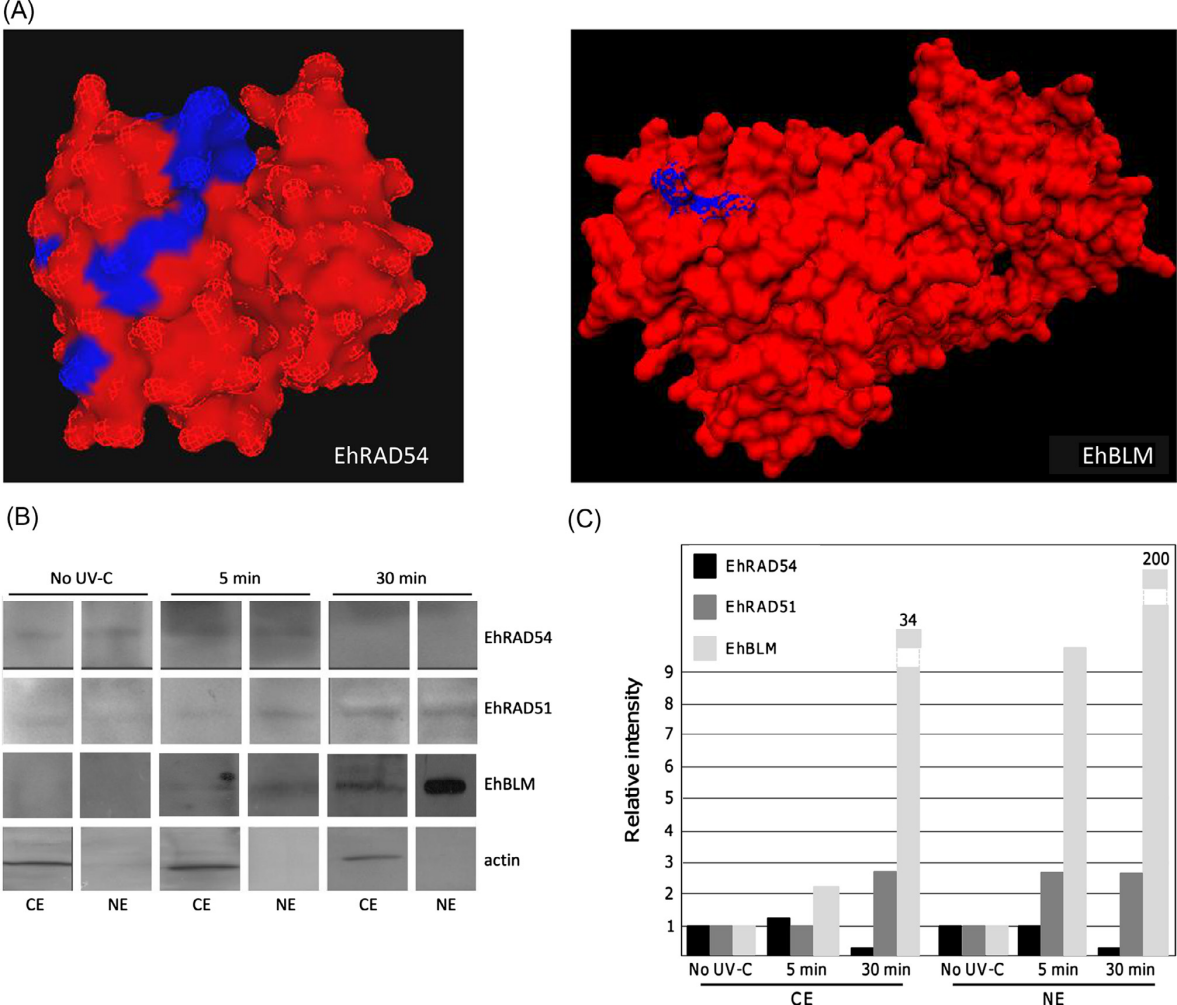
Immunodetection of EhRAD54, EhRAD51, and EhBLM. (A) Localization of EhRAD54-pepA and EhBLM-pepB peptides in the 3D model of each protein. (B) Western blot assays of cytoplasmic (CE) and nuclear (NE) extracts from trophozoites without irradiation (No UV-C) or at 5 min or 30 min after DNA damage. The name of each inmmunodetected protein is indicated at the right. Actin was used as control. (C) Densitometry analysis of bands revealed in (B). Pixels corresponding to cytoplasmic actin were taken as 100% at each time after DNA damage, and used to normalize data. The relative expression of each protein is expressed using the protein amount before UV-C exposure as a reference. Results in (B) and (C) are representative of three replicates.

To gain insights into the molecular events underlying HR in *E. histolytica*, we next analyzed the expression of EhRAD51, EhRAD54, and EhBLM in response to DNA damage by Western blot assay. EhRAD54-pepA (KPGILEVSFDKLLLF) and EhBLM-pepB (KKASKKSTNSSSNG), corresponding to regions spanning 92–106 amino acids and 1164–1177 amino acids in EhRAD54 and EhBLM proteins, respectively, were chosen as antigenic peptides. Interestingly, both peptides are located in a region that is exposed to solvent in the predicted 3D structure of EhRAD54 and EhBLM proteins, respectively (Figure 2A). Specific EhRAD54 and EhBLM antibodies generated as described in “Materials and Methods” section and EhRAD51 antibody previously obtained [14] were used to analyze protein expression in trophozoites in response to UV-C irradiation. As shown in Figure 2B and C, EhRAD54 was detected in both CE and NE obtained from non-irradiated trophozoites; EhRAD54 expression was maintained at 5 min after DNA damage while it decreased drastically at 30 min. On the other hand, EhBLM detected in both CE and NE obtained from non-irradiated trophozoites increased more than nine-fold in NE at 5 min after irradiation. Importantly, at 30 min after DNA damage, we observed a higher increase in EhBLM expression, mainly in NE (200-fold). EhRAD51 was expressed at 5 min after DNA damage, and even more at 30 min. EhRAD51 was expressed at 5 min after DNA damage, and even more at 30 min. These results are consistent with our previous report, showing a dramatic increase in EhRAD51 in the cytoplasm and nucleus, at 30 min after DNA breaks were introduced into the *E. histolytica* genome. Intriguingly, we did not observe the molecular weight change in EhRAD51 that has been postulated to correspond to some posttranslational modifications of the cytoplasmic protein for its translocation to the nucleus where DNA repair takes place [14]. Pre-immune sera tested as control did not give any signal (data not shown).

The kinetics of expression of EhRAD54, EhRAD51, and EhBLM proteins in *E. histolytica* suggests that the recruitment of these proteins in response to DNA DSBs occurs at the same steps of HR as described in humans and yeast, indicating the conservation of HR events through evolution [4]. Therefore, based on the results described here and previously [14], we propose a hypothetical model to describe the DNA DSB repair steps by HR in *E. histolytica* trophozoites. At 5 min after DNA damage, EhRAD54 may be recruited to DSB sites to contribute to chromatin remodeling together with phosphorylated EhH2AX histones. EhRAD54 may also stabilize the nucleofilament formed by EhRAD51 bound to ssDNA to promote DNA pairing and homology search. In addition, EhBLM may regulate chromatin organization at DSBs and EhRAD51 nucleofilament dissociation at this time. Later, at 30 min, EhBLM may participate in events related to branch migration and D-loop unwinding.

In conclusion, our results showed that the predicted EhRAD54 protein conserves the molecular features that are characteristic of homologous proteins in humans. Moreover, the differential expression of EhRAD54, EhBLM, and EhRAD51 proteins in response to DNA damage confirmed their potential roles in HR in this deep-branching eukaryotic parasite. Further experiments to evaluate protein-protein interactions and protein activities should help to understand the specific roles of each protein in DNA repair by HR in *E. histolytica*.
